# Favouring respiration over locomotion in chronic obstructive pulmonary disease

**DOI:** 10.1113/EP092731

**Published:** 2025-07-31

**Authors:** Kiana M. Schulze, David C. Poole

**Affiliations:** ^1^ Department of Kinesiology Kansas State University Manhattan Kansas USA; ^2^ Department of Anatomy and Physiology Kansas State University Manhattan Kansas USA

1

Much like Dr. Pangloss, Candide's teacher and philosopher in Voltaire's (1694–1778) satirical novella (Voltaire, 1975), past American Physiological Society President (1914–16) and apex physiologist Walter B. Cannon (1871–1945) crystallized the notion in his ‘wisdom of the body’ ethos that physiology and physiological adaptations arc towards homeostasis and the good, or at least survival, of the organism. In this arena, the editorial by Hartmann, Caldwell, et al. (2026) asks whether the divergent patterns of muscle fibre adaptation seen in the diaphragm versus locomotory skeletal muscles (e.g., quadriceps) in response to emphysema or chronic obstructive pulmonary disease (COPD) are helpful (adaptive) or harmful (maladaptive).

The acute physiological response to intense cold exposure, whereby the body vasoconstricts the skin and periphery, demonstrates that sacrifice of the extremities to frostbite can be necessary for survival of the organism. Likewise, in heart failure with reduced ejection fraction, the massive kidney‐driven sodium retention, volume expansion, systemic inflammation and muscle vasoconstriction are pernicious collateral damage invoked to ensure that the renal blood flow is preserved. These two exemplars challenge the concept that adaptive responses are all necessarily ‘good’.

Even in healthy individuals, the diaphragm can fatigue and, with the continuously increased energetic demands of COPD/emphysema, the likelihood of diaphragmatic failure is exacerbated (Roussos & Macklem, 1977; reviewed by Levine et al., 2002). Thus Hartmann, Caldwell, et al. (2026) effectively document how, in response to the chronically imposed greater energetic demands of COPD (mechanical disadvantage, impaired lung function and greater ventilatory requirements), the diaphragm undergoes a transformation from type II to more oxidative and fatigue‐resistant type I fibres. Simultaneously, however, the quadriceps muscles transform to a less oxidative, more fatigable phenotype, with decreased O_2_ diffusion capacity and less ability to increase perfusive O_2_ conductance in response to exercise and, potentially, exercise training (reviewed by Broxterman et al., 2021; Hartmann, Caldwell, et al., 2026). This pattern of adaptation again demonstrates that, in COPD as in cold exposure and heart failure, there is a hierarchy of preserved function with the essential role of the diaphragm to preserve gas exchange, and thus life, selected over peripheral skeletal muscles. This behaviour is seen acutely during experimentally increased respiratory muscle work (Harms et al., 1998) and chronically in COPD (reviewed by Hartmann, Caldwell, et al., 2026), where the diaphragm will ‘steal’ blood flow, and therefore O_2_ supply, from the active locomotory muscles. Importantly, respiratory muscles (e.g., diaphragm, external and internal intercostals, scalenes, abdominals) can contribute in various degrees to the work of breathing that can be differentially impacted by COPD (versus health) (Sexton & Poole, 1998; Vogiatzis et al., 2020) and thus, investigating blood flow distribution among multiple respiratory and locomotory muscles across a range of ventilatory demands is warranted.

Appropriately, Hartmann, Caldwell, et al. (2026) address the likely importance of capillary (mal)adaptations in the locomotory skeletal muscles that might contribute to impairments in both perfusive and diffusive O_2_ conductances, and thus limit exercise tolerance in COPD. In this regard, Broxterman et al. (2021) found that small muscle mass exercise training could improve muscle maximum O_2_ uptake primarily via elevated diffusive, but not perfusive, conductance. Intriguingly, a separate study by Hartmann, Nymand et al. (2025) demonstrated that whole‐body high‐intensity exercise training primarily increased skeletal muscle perfusive conductance in COPD. Together, these studies raise the interesting possibility that a combination of small and large muscle mass exercise training might potentiate improvements in exercising locomotory muscle perfusive and diffusive O_2_ conductances. It would be valuable to determine the mechanistic bases for these effects by observing microvascular red blood cell flux and distribution within the locomotory muscles during exercise, although this is technically infeasible in humans at present. It is pertinent that, at least in the rat, fast‐twitch muscles sustain a far lower microvascular O_2_ partial pressure (Colburn et al., 2020) versus slow twitch, in addition to lower energetic efficiency, both of which are conducive to greater fatigability as found in locomotory muscles in COPD.

Overall, Hartmann, Caldwell, et al. (2026) outline a compelling case for positive adaptations within the diaphragm acting to increase oxidative function and fatigue resistance in COPD. Sadly, this occurs concomitantly with impaired locomotory muscle function which might be redressed effectively by judicious selection of small or large muscle mass training, or a combination of both, depending on the capabilities of the specific patient.

Hartmann, Caldwell, et al. (2026) present the dilemma of whether progression of the disease leads to reduced physical activity and locomotory muscle dysfunction in COPD or whether muscle dysfunction comes first. Answering this question is crucial to unravelling the mechanistic bases for skeletal muscle dysfunction in COPD. However, whichever scenario is proved correct, it is a clinical and societal imperative to develop effective strategies to improve exercise tolerance and quality of life and to reduce morbidity and mortality in these patients.

Hartmann, Caldwell, et al. (2026) liken the adaptive response to COPD in terms of Aristotle's paradox: Which came first, the chicken or the egg? At least in this regard, logic and subsequent scientific progress can help. In the late 19th century, before Mendelian genetics (Gregor Mendel, 1822–1884) and Darwinian evolution (Charles Darwin, 1809–1882) were widely accepted, Lamarckian theory (Jean‐Baptiste Lamarck, 1744–1829) held sway. Specifically, Lamarck considered that anatomical alterations, for instance, an amputated limb, incurred by a parent could be transmitted to the offspring. However, in 1888, following a decisive series of studies amputating the tails of generations of mice and failing to detect any tail shortening in offspring, August Weisman (1834–1914) concluded that acquired characteristics were not transmissible across generations (reviewed by Weisman, 1890). Accordingly, two birds that were, by definition, not chickens mated, and their union formed the egg containing a chicken. Hence, the egg came first!

The question regarding the proximate cause of the dysfunctional leg skeletal muscles in COPD patients highlighted by Hartmann, Caldwell, et al. (2026) is pressing and complex, in part owing to the broad heterogeneity of COPD phenotypes. However, given the crucial role of physical activity in restoring quality of life and reducing morbidity in COPD and other chronic diseases, it is imperative that it be addressed.

## AUTHOR CONTRIBUTIONS

Kiana M. Schulze and David C. Poole: conceptualization, writing, and editing. Both authors approved the final version of the manuscript and agree to be accountable for all aspects of the work in ensuring that questions related to the accuracy or integrity of any part of the work are appropriately investigated and resolved. Both persons demarcated as authors qualify for authorship, and all those qualifying for authorship are listed.

## CONFLICT OF INTEREST

None declared.
